# Voices that matter: end-of-life care in two acute hospitals from the perspective of bereaved relatives

**DOI:** 10.1186/s12904-018-0365-6

**Published:** 2018-10-19

**Authors:** Sarah Donnelly, Geraldine Prizeman, Diarmuid Ó Coimín, Bettina Korn, Geralyn Hynes

**Affiliations:** 1 School of Social Policy, Social Work and Social Justice, University College Dublin, Belfield, Dublin 4, Ireland; 2 Trinity Centre for Practice and Healthcare Innovation, School of Nursing and Midwifery, Trinity College Dublin, 24 D’Olier Street, Dublin, D02 T283 Ireland; 3 Mater Misericordiae University Hospital, Quality and Patient Safety Directorate, Eccles Street, Dublin 7, Ireland; 4 1st Floor CEO Building, St. James’s Hospital, James Street, Dublin 8, Ireland

**Keywords:** End-of-life care, Palliative care, Acute hospital, Quality of care, Bereaved relatives, Qualitative, VOICES, Bereavement, Dying

## Abstract

**Background:**

End-of-life care (EoLC) is an experience that touches the lives of everyone. Dying in an acute hospital is a common occurrence in developed countries across the world. Previous studies have shown that there is wide variation in EoLC and at times is experienced as being of poor quality. Assessing and measuring the quality of care provided is a key component of all healthcare systems. This paper reports on the qualitative analysis of open-ended free text questions that were asked as part of a post-bereavement survey conducted in two adult acute hospitals in Ireland.

**Methods:**

This was a quantitative descriptive post-bereavement postal survey, gathering data retrospectively from relatives or friends of patients who died, utilising an adapted version of the VOICES (Views of Informal Carers - Evaluation of Services) questionnaire. *VOICES MaJam* has 29 core questions, seven questions requesting personal demographic information and four open-ended questions gathering descriptive data about the care experience during the patient’s last admission to hospital. A total of 356 valid questionnaires were returned. Qualitative data were managed, coded and analysed with NVivo 10, using a template analysis framework approach.

**Results:**

Three quarters (75%: *n* = 268) responded to at least one of the open-ended questions. Several key themes emerged, indicating areas that require particular attention in EoLC. Two themes relate to how care needs are met and how the hospital environment has a critical influence on EoLC experiences. The remaining three themes relate more to the interpersonal context including whether patients are treated with dignity and respect, the psychological, emotional and spiritual needs of patients and their family members and communication.

**Conclusions:**

Acute hospitals need to ensure that patients and their relatives receive high quality EoLC. Seeking the views of bereaved relatives should be considered by all hospitals and healthcare settings to ascertain the quality of care at end of life. This study contributes to our understanding and knowledge of what good EoLC looks like and where care can be improved, thus enabling hospitals to direct and inform quality improvement.

## Background

End-of-life care (EoLC) is an experience that touches the lives of everyone. For many seriously ill people across the world, dying in an acute hospital is a common occurrence [1–3]. A cross-national comparison of location of death indicated that across 40 populations, all deaths occurring in hospitals were reported to be between 40 and 60% [4]. Despite many people expressing a preference to die at home [5]_,_ research has indicated that four in ten (43%) of all people in Ireland die in an acute hospital with 6% dying in a hospice [6].

Previous studies have shown that there is wide variation in the quality of EoLC in acute hospital settings [7–12]. One of the key factors identified as contributing to sub-optimal care is a lack of open communication particularly, a lack of information regarding the condition and prognosis of the person who died [13, 14]_._ In addition, often basic nursing care needs and symptom control are not adequately managed [7, 9, 15]. It is generally accepted from studies carried out to date that good care at end of life is characterised by the careful management of pain and other symptoms, patient dignity and independence, family involvement and support, awareness of death and good communication with healthcare professionals [16–20]. The hospital’s physical environment is also recognised as an important factor in the care of those seriously ill and at end of life [3, 16, 21, 22]. These factors reflect the multifaceted and complex nature of EoLC which encompasses physical, medical, social, spiritual and psychological experiences [17, 23, 24].

Acute hospitals are fast paced environments with a predominant focus on diagnosis, treatment and cure, however, caring for people who are seriously ill is also an important responsibility. Over the last decade there have been a number of initiatives addressing EoLC experiences in acute hospitals in Ireland supporting the work of specialist palliative care. These include a national audit of EoLC [25], the development of quality standards for EoLC [26], the Hospice Friendly Hospital programme [27, 28] and the appointment of EoLC coordinators in some hospitals. The importance of capturing the patient experience has also been stressed; “*If quality and safety is at the heart of everything we do, we must understand it from the perspectives of our service users”* [29, 30]. These initiatives have helped to drive an increasing focus on examining and improving all issues related to EoLC experiences of patients and their relatives/carers.

Assessing and measuring the quality of care provided is a key component of all healthcare systems [14]. However, this poses extra difficulties considering the sensitivities and ethical issues that arise in the area of EoLC. Surveying patients who are “likely to die” is challenging given the difficulties associated with predicting death, the significant ethical issues involved and the burden it would place on the dying person [31]. Given the complexities and sensitivity of collecting data from dying patients, the use of family members as proxies has been found to be an adequate substitute [14, 31, 32]. For this reason, studies have primarily focussed on ascertaining the views of family members to give “voice” to their experience of care and that of the person who died. Of significance is that surveying bereaved relatives is increasingly recommended to ascertain the quality of EoLC in healthcare settings [14, 33–36]. This paper reports on the qualitative analysis of four open-ended free text questions that were asked as part of a post-bereavement survey conducted in two adult acute hospitals in Ireland [37].

End-of-life Care is defined and interpreted in literature and policy internationally in various ways from care in the last days and hours of life [36] to a broader interpretation, to the care provided to people who are likely to die within 12 months such as people with incurable and life-limiting conditions and those who die unexpectedly. It also refers to care provided to relatives [25]. The latter definition of end-of-life care is utilised for the purposes of this paper.

Research was undertaken in two large adult acute university teaching hospitals in a city setting. Both hospitals provide acute services for their catchment area, and are tertiary centres, providing a range of frontline and specialist services on a regional and national level. Both hospitals have specialist palliative care teams consisting of palliative medicine consultant, registrar, clinical nurse specialists and social worker. The hospitals are also active members of the Hospice Friendly Hospital Programme [28] and each hospital has an EoLC Co-ordinator who is tasked with leading, supporting and coordinating all activities associated with implementing *Quality Standards for End-of-Life Care in Hospitals* [25]. In addition, each hospital has an EoLC Committee with representatives from clinical, administrative and support services across each hospital with a focus of improving all aspects of end-of-life care.

## Methods

### Background and design

A quantitative descriptive post-bereavement study which gathered data retrospectively, using a postal survey, from relatives or friends of patients who died in two acute hospitals [37]. An adapted version (*VOICES MaJam*) of the VOICES (Views of Informal Carers – Evaluation of Services) questionnaire [38] was utilised. VOICES is a validated post-bereavement survey and focusses on those aspects of care which are known to be indicative of the quality of care for patients nearing end of life and their families. The VOICES questionnaire was adapted to reflect the context of acute hospital care in Ireland to ascertain if the respective hospitals were meeting the principles of care outlined in key policy documents. *VOICES MaJam* has 29 core questions and an additional seven questions requesting personal demographic information. In addition, four open-ended questions were included to gather descriptive data about the care experience during the patient’s last admission to hospital.

### Sample selection

Persons recorded as the next of kin in the deceased person’s healthcare record were recruited for the sample. Relatives of people who died from August 1st 2014 to January 31st 2015 were included. The sample included relatives who were bereaved no earlier than 3 months and no later than 9 months. All deaths, including sudden and unexpected deaths were included. The exclusion criteria for this study included the following; patients aged less than 18 years of age; patients who did not die in the hospitals; and, next of kin with a missing or incomplete address. The combined sample size was 792 (Hospital A: *n* = 385; Hospital B: *n* = 407).

### Data collection

Data were collected in three waves between May and September 2015. A study information sheet, opt-out slip and information on bereavement supports were included with the survey pack. Respondents were informed that return of the questionnaire was viewed as consent to participate. Relatives were also provided with contact details of the principal investigator in each hospital if they had any queries or concerns about the study. A total of 356 valid questionnaires were returned (Hospital A; *n* = 167: Hospital B; *n* = 189), giving an overall response rate of 46%.

Three quarters of relatives (75%: *n* = 268) responded to at least one of the four open-ended questions. Three quarters (75%: *n* = 268) commented on some aspect that was good about the care. Forty-seven percent (*n* = 166) responded to the second question, *What, if anything, do you feel was bad about the care?* However, 58 (16%) of those who answered this question indicated clearly that they experienced nothing bad about the care their relative received. Finally, 163 (46%) relatives provided additional comments on the care provided by the staff during their relative’s last admission to hospital.

The findings being reported on in this paper are based on the 268 relatives who responded to at least one of the following open ended questions:

- *What, if anything, do you feel was good about the care?*
- *What, if anything, do you feel was bad about the care?*
- *Please use the space below if there is anything more you would like to add about the care provided by the hospital to your relative/friend during their last admission.*
- *Is there is any other help or support that you would have liked to receive from the hospital since your relative’s death, please feel free to comment below.*

### Data analysis

**Fig. 1:**
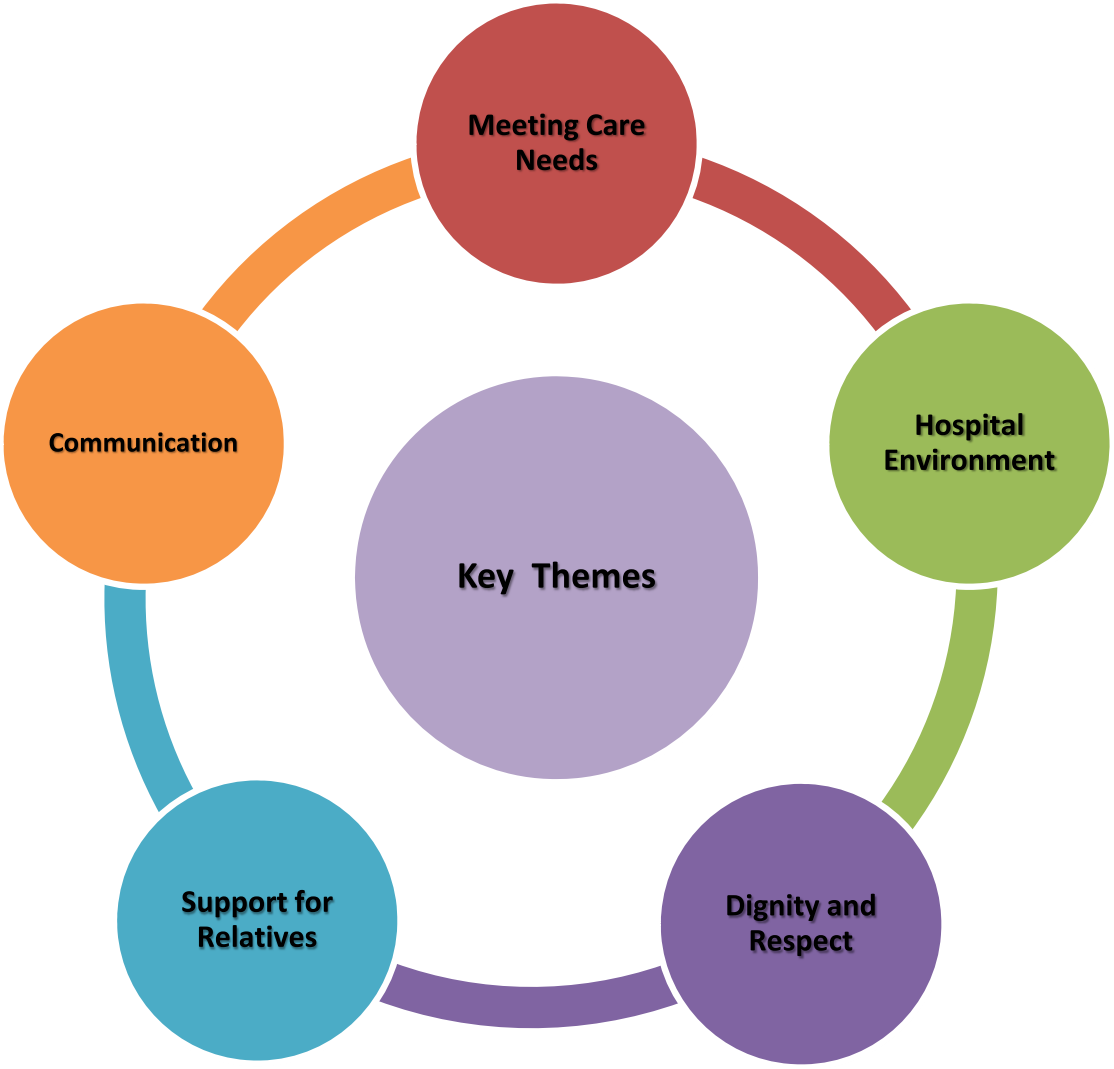
Key Themes

Qualitative data were managed and coded using NVivo 10 [39]. A coding frame was developed based on the principles and standards of care outlined in the *National Healthcare Charter* [29] *National Standards for Safer Better Healthcare* [40] and *Quality Standards for EoLC in Hospitals* [25] (See Table 1). Data were analysed thematically using a template analysis framework approach [41]. Five key themes emerged: communication, meeting care needs, hospital environment, dignity and respect and support for relatives (see Fig. 1: Key Themes).

**Table 1:** VOICES *MaJam* Coding Frame

Node No.	Parent node	Child node	Definition/ description of terms
1	Person-centred	1.1 Kindness & Compassion (Positive) 1.2 Dignity & Respect (Positive) 1.3 Kindness & Compassion (Negative) 1.4 Dignity & Respect (Negative)	Staff attitudes (incl. Mention of professionalism) Caring Attentiveness Going extra mile Being understanding Empathy
2	Patient preference	2.1 Positive Comments 2.2 Negative Comments	Preferred place of care, of treatment, etc. Person centred approach to patient preferences
3	Equity of access	3.1 Positive Comments 3.2 Negative Comments	To services (having to come through A/E where others don’t as perceive by respondent) to palliative care team/other specialists or community services, etc. (examples include comments on weekends and out-of-hours services)
4	Safe practice/environment	4.1 Staff skills (positive) 4.2 Staff shortages (positive) 4.3 Safe environment (positive) 4.4 Staff skills (negative) 4.5 Staff shortages (negative) 4.6 Safe environment (negative)	Competency to manage symptoms/deliver care e.g. A/E trolleys, over-crowding, other patients. Including mentíons of general care.
5	Good Communication	5.1 Patient 5.2 Relative 5.3 MDT	Being informed/being able to express concerns; responsiveness of staff, being asked about concerns/needs/ Communication a two-way process Verbal & nonverbal & written communication aspects Incl. mentions of bereavement card/letter
6	Poor Communication	6.1 Patient 6.2 Relative 6.3 MDT	Being informed/being able to express concerns; responsiveness of staff, being asked about concerns/needs/ Communication a two-way process Verbal & nonverbal & written communication aspects
7	Shared decision making/ participation	7.1 Positive Comments 7.2 Negative Comments	Patient and/or relative and staff (participation for patient and/or relative)
8	Privacy	8.1 Positive Comments 8.2 Negative Comments	Overheard/exposure Personal space/public place
9	Symptom management	9.1 Physical (positive) 9.2 Psychological (positive) 9.3 Social (positive) 9.4 Emotional (positive) 9.5 Spiritual (positive) 9.6 Physical (negative) 9.7 Psychological (negative) 9.8 Social (negative) 9.9 Emotional (negative) 9.10 Spiritual (negative)	Psychological = depression Social Emotional = upset Spiritual (may be religious/humanist)
10	Physical environment	10.1 Positive Comment 10.2 Negative Comments	Facilities on wards/hospital (e.g. refreshments)/ parking/mortuary/ family room/single room/cleanliness/visiting times
11	Family support	11.1 Support presence of family (positive) 11.2 After death care (positive) 11.3 Bereavement care (positive) 11.4 Support presence of family (negative) 11.5 After death care (negative) 11.6 Bereavement care (negative)	Physical, psychological, social, emotional, spiritual Sensitive/appropriate Incl. mention of bereavement card/letter
12	Coordination of care	12.1 Across teams (positive) 12.2 Within MDT (positive) 12.3 Across teams (negative) 12.4 Within MDT (negative)	Chaos Calling the shots
13	Patient care needs	13.1 Prior to death (positive) 13.2 After death (positive) 13.3 Prior to death (negative) 13.4 After death (negative)	Basic physical care needs (comfort, positioning, intake) e.g. Laying out of the deceased
14	Post Mortem		Issues around process of investigation of cause of death
15	Nutrition		Food intake not being monitored Specialist diet requirements not being met e.g. Parkinson’s
16	Additional help and support (Q29)	16.1 Person-centered 16.2 Patient preference 16.3 Equity of access 16.4 Safe practice/environment 16.5 Good communication 16.6 Poor communication 16.7 Shared decision making/participation 16.8 Privacy 16.9 Symptom Management 16.10 Physical environment 16.11 Family Support 16.12 Coordination of care 16.13 Patient care needs 16.14 Post Mortem 16.15 Nutrition 16.16 Access to palliative care 16.17 Methodology 16.18 General Comments	Q29 very specifically worded about additional help and support that relative would have liked – need to capture these responses separately so code here.
17	Access to palliative care		
18	Methodology	18.1 Helpful or beneficial 18.2 Thankful for the opportunity	
19	General Comments

### Rigour

Inter-rater reliability tests were conducted by two independent researchers, indicating a kappa score of 0.62. In addition, the research team consisted of an EoLC Co-ordinator from each hospital and experienced researchers from their respective academic partners providing a unique balance of practice and academic expertise.

The Research Ethics Committees in the respective hospitals granted full ethical approval for the study.

## Results

### Demographics and overview

Three quarters of respondents were female (75.3%: *n* = 201) and four in ten (40.7%: *n* = 108) were over the age of 60 years. Children of the deceased were the largest group of respondents (44.3%: *n* = 117), followed by husband (18.9%: *n* = 50) or wife (13.6%: *n* = 36), including civil partner. Over half (57.4%: *n* = 151) of the deceased were male, with (51.9%: *n* = 138) being over the age of 80 years. Almost three in ten (28.8%: *n* = 74) of the people that died, had a stay of between 2 days and 2 weeks and one fifth (20.2%: *n* = 52) had a stay of longer than 2 months.

### Communication

This theme centred on good and poor communication and explored relatives’ experience of communication with the patient and family and within the multidisciplinary team. Many relatives highlighted good communication practices both by individual staff members but also within and across multidisciplinary teams to patients and their family members:

*“The care and attention shown to my brother was excellent. At all stages of his illness we were kept informed and that was important to me, my wife and family.”*

Other relatives reported that staff communicated in a timely, sensitive and compassionate manner both to patients and their family members, this was a great source of comfort to them:

*“There was always someone on duty to explain to us what was happening at all stages of his illness and eventually his death. This is a great comfort when you know your loved one is about to die. And it helps ease the pain of knowing they are going to pass away.”*

Some relatives reported poor communication however, including lack of sensitivity and compassion by hospital staff.

*“The Dr taking care of my mother’s needs, came into the room where she was lying still alive, and spoke over her bed telling me that there was no more they could do for her and they made her comfortable. My mother was still awake and listening to all this. I think maybe he should have called me outside to tell me this.”*

Lack of communication or information being conveyed in what was deemed an inappropriate manner was also reported:

*“…staff never appeared to have any time, a small number of staff took time to talk to my Dad, no conversation appeared to have been about his diagnosis/illness. I appreciate there are a lot of constraints on staff but surely it’s important to get the basics right.”*

Relative’s comments indicated that communication of information in a supportive, timely and regular manner could be improved in EoLC. Interestingly, the issue of communication threads through most of the other themes in this study indicating that communication significantly impacts on all aspects of EoLC.

### Meeting care needs

A large amount of data was coded under this theme, resulting in the emergence of several subthemes including the provision of personal care, staff skills and availability, pain and symptom management, emotional and psychological support, spiritual support and access to specialist palliative care outside normal working hours and at weekends.

Many relatives commented specifically on the skill level of staff members and particular mention was given to staff from different disciplines. Their skills, compassion and dedication were highlighted:

*“As I’ve said throughout this survey I am amazed by the dedication, empathy and patience of doctors, nurses and ancillary staff…My family could not fault our experience at all.”*

The high skill level and dedication of staff left a significant impression on relatives. Findings illustrate that all staff, whom patients and families come into contact with, have a critical influencing role in a patient’s end-of-life journey and care. The positive impact of therapeutic relationships between staff and patients were also emphasised by relatives:

*“… we had the opportunity to build relationships with the staff there and they built a relationship with my dad.”*

While many relatives praised the high skill level of staff and their ability to meet care needs, some relatives indicated that care could be improved. Many relatives indicated that while the patient’s pain was well managed, and the patient was well cared for, all care needs did not always seem to be fully met. Relatives cited staff shortages as impacting on the provision of patient care. Relatives indicated that the management of psychological and emotional needs, in line with patient preferences, at end of life could be improved. In addition, spiritual care was seen as an important aspect of EoLC that was perhaps not always fully considered and responded to by hospital staff. One relative noted the importance of the emotional support provided by hospital staff and the positive impact this had on the family:

*“Nurses, carers, staff and doctors were truly excellent with our father. Their genuine concern and tenderness was above the call of duty … the carers maybe just held his hand and consoled him and told him things would be OK. This meant so much to my family…we will be forever in the nurses and staff debt…”*

However, several of the comments relatives made about the management of emotional needs were negative in tone and referred to ways in which hospital staff failed to meet patient care needs and adequately address relatives’ concerns.

Several relatives commented on the spiritual care within the hospitals. Those that shared a positive experience, referred to the important role played by the Chaplain or pastoral care worker in providing spiritual care to patients at the end of life and the clear recognition of patients’ needs in this respect. A small number of relatives shared their negative experiences of spiritual care during the patient’s last admission. The emphasis here was on the lack of consideration of the individual patient’s wishes regarding spiritual and religious matters. In addition, the importance of spiritual care for some patients was not always recognised by hospitals or staff: *“Maybe there could be a little more focus on spiritual needs for patients across all cultures and a proper room or oratory available to all.”*

Many relatives also commented on palliative care provided to their relatives. Some relatives had an extremely positive experience of the specialist palliative care team involvement and valued the unique skills which this team could offer:

*“When the palliative care team in the hospital joined forces with it all, it was even better. They got her back into pyjamas she was less a patient, more cared for as a human being who was very ill.”*

While the involvement of the specialist palliative care team was welcomed, some relatives expressed surprise and dissatisfaction that there was no access to specialist palliative care at the weekends:

“*I found it strange that the “hospice team” did not work weekends!!! This makes no sense at all. My mother died on a Sunday and we could have done with their support instead we had a weekend staff who were awkward dealing with the situation.”*

### Dignity and respect

Most relatives indicated that they and the person who died were treated with dignity and respect and experienced kindness and compassion. However, some families reported negative experiences. Relatives referred to the impact the dignity and respect shown to patients had on their overall hospital experience. One respondent reported:

*“The most impressive aspect was the degree to which the team made us feel that our Mother’s wellbeing was important, that our feelings were respected and there was a real sense of staff caring and not viewing the situation as “one of many cases” – which of course it was.”*

Other relatives had differing experiences and for some they believed dignity and respect was lacking in the care provided:

*“We argued that she as a person deserved her dignity and privacy around herself in the last hours of her life. We also felt that it was not fair to either her large family or the other ward patients that my mother’s final hours be lived out on a hospital ward.”*

There were many positive comments provided about occasions where kindness and compassion were displayed to patients and their relatives. These often related to the attitude of staff, their professionalism, interpersonal communication and the ways in which they engaged with patients, their families and friends:

*“He was called by his name, his name was used when carrying out any procedure, even though some of the time he was unaware. The staff ... were kindness itself. We can never underestimate the power of kindness.”*

### Hospital environment

Findings in this theme fell under four sub-themes: care in a single room, hospital facilities, route of admission and atmosphere in the ward. Interestingly, privacy was viewed as an overall key factor influencing a patient’s end of life experience. The importance of having access to a single room was repeatedly emphasised as making for a better overall hospital experience:

*“As mam deteriorated she was transferred to a private room with an ensuite. Here we were offered as much access to mam and could stay overnight if we wished.”*

Other relatives advised that as they were in a shared ward, they had to be mindful of other patients and this lack of privacy was distressing for them. Some relatives, who were unable to access a single or family room, described how this significantly impacted on their relatives’ experiences in a negative way in the days before they died. One relative commented on the noise in the ward made by staff and other visitors on the day of their family member’s death:

*“During the day, when mam was in effect dying I found it hard to hear other people’s visitors laughing and Hoovers, cleaners shouting…We were really hoping that we could have a private room.”*

Some relatives questioned the accessibility of the hospital for those receiving end-of-life care. Relatives also reported that access to beds for terminally ill patients was often problematic, particularly as the only process for admission to hospital, even for patients who were well known to the hospital, was via the Emergency Department. This experience was described as extremely stressful and relatives deemed the Emergency Department as being an unsuitable environment for terminally ill or older patients to await admission to hospital:

*“As he …was diagnosed as a terminally ill patient, he should have been seen straight away by a doctor from the team that looked after him. I think the hospital should really consider their procedures surrounding admittance of terminally ill patients.”*

Other relatives shared more positive experiences about the suitability of the hospital facilities, mainly referring to the general atmosphere in the ward or room as *‘ideal’* or *‘peaceful’.*

The importance of having access to a family room on the ward to facilitate difficult, sensitive conversations in private with the medical team was commented on by many:

*“The consultations about the condition of the patient were very informative. We did not like the fact that the consultations were held in the corridors, this was not the best or most comfortable place but the doctors could only use what space was available.”*

Relatives identified access to a family room and overnight accommodation as a crucial resource, which greatly enhanced their care experience. Having access to a family room was *‘useful’* or *‘vital’* allowing privacy and unrestricted visiting for which families were extremely grateful:

“*We were hugely grateful for the use of the family room and for the chance to be with my husband as a family in his final days. As a large family, our presence in the ward was tolerated in a very compassionate manner for the most part by the nursing staff. For this we are extremely grateful.*”

Other relatives acknowledged the importance of having access to a family room but highlighted the need for adequate facilities in the room, such as tea and coffee making facilities and a bright, comfortable environment:

*“[The] Family room is a benefit but in this case the room was full of old furniture and not very comfortable and two families waiting at a time which limited privacy.”*

Many stressed the need for accessible, dedicated family rooms with appropriate facilities on all hospital wards to support the EoLC experience for patients and their family members.

### Support for family members

Relatives suggested several ways during EoLC where they believed additional support for family members could be provided during the patient’s stay in hospital. These included;

1. Privacy and sensitivity;
2. Unrestricted visiting;
3. Affordable parking and ease of access;
4. Support at the time of death;
5. Bereavement support
6. Support from the social worker; and
7. Support from taking part in the survey.

During the patient’s stay in hospital, relatives appreciated the “*courteous and sympathetic attention”* that was given and being allowed to spend precious time with their sick relative. Other relatives drew attention to situations where staff displayed great sensitivity and thoughtfulness:

*“The doctors and nursing staff were very sensitive when telling us the difficult news that my mother was going to die. The staff in [name of ward] particular were amazing and as a family it was helpful to be with mum the whole time. We were very lucky that my mother was given a room of her own...”*

Other relatives reported struggling with knowing how to talk to their family members about the fact they were dying and stated they would have appreciated support and advice from staff in relation to this:

“*I did not know who to approach re: telling my husband he was dying, I knew years ago the ward sister was the guide for this. I did seek advice from one staff member … her most unhelpful answer was “just ask him if he has anything he wants to say to you*.”

The issue of unrestricted visiting was raised by relatives and their comments emphasised the stress and frustration felt by family members who were “*not given the opportunity to visit outside of visiting hours.”* Out of hours visiting for relatives is permitted in both hospitals when a patient is seriously ill or dying, however, several relatives were not made aware of this. Relatives reported problems with the financial cost of car parking which caused additional stress at what was already a very difficult time for them. They suggested that the cost of parking in hospitals was too expensive:

*“The cost of parking was outrageous. Given that we were there for 11 weeks, it would be nice if family members could get some help with this cost.”*

Feedback from relatives about their experiences after the death of their loved one were mixed. Relatives described the support they experienced in differing ways. One commented that:

*“They laid my father out lovely, gave us a lovely room with candle and flowers and gave us as much time and space as we needed but yet were there if we needed them or wanted to ask any questions.”*

Another relative had a less positive experience and reflected on the upset caused by not being able to view their relative’s body in the hospital mortuary over the weekend, when release of remains were delayed preventing funeral arrangements to proceed.

Some relatives were appreciative of the sympathy displayed by the hospital whether in person, by a letter or card or remembrance service:

*“Receiving a hand written sympathy card from the sister [nurse manager] in charge of his care was very special. It was personal and comforting.”*

Other relatives commented on having accessed bereavement counselling offered and that they found this support extremely helpful. One relative described what she thought was “*exceptional*” bereavement support that she received:

*“I have to say that the aftercare that I received when I arrived at the hospital was exceptional, especially from the nursing staff. I was contacted the next day by a bereavement counsellor who was more than helpful and continues to support me 6 months later. Outstanding work by all in an extremely difficult situation.”*

Several relatives highlighted bereavement support that they would have liked to receive from the hospital. These included;

- Timely bereavement information
- Follow up contact from the bereavement counsellor in the hospital (for example, 3 months’ post-bereavement)
- Provision of a memorial service
- Provision of links to people who have experienced a similar bereavement
- Information regarding supports and financial entitlements available to the bereaved.

A subtheme to emerge was that of the important role of social work in providing support to family members during this time. Many relatives acknowledged the beneficial help and support which the medical social worker was able to offer and the useful role of family meetings in providing information and facilitating decision making. Relatives appeared to have mostly positive experiences when they could access support from a social worker. Some relatives however, were unhappy that they were not able to access help from a social worker, in a timely manner when they felt they really needed it:

*“I did not get the help I needed at the proper time – no social worker...I live alone, no relatives. Had to do it all myself, with the help of a friend.”*

An unexpected finding was that relatives spoke about the cathartic process of completing the survey. It gave relatives a ‘voice’ to describe their experiences, provided an opportunity for reflection and many indicated that they gained support from taking part in the survey. Some relatives appreciated the opportunity to express their gratitude to the hospital for their *“dedication and professionalism”* while others used the opportunity to *“express their feelings”* about aspects of EoLC provided to them and their relatives. It appears that some relatives welcomed the questionnaire as an opportunity to report on their experiences and that of their relative who had died. It was viewed as an opportunity for their voices to be heard on their end-of-life care experience:

*“I understand that this survey has little to do with the prescribed treatment of our mother and how it affected her. It is however the only platform we have had so far to state our feelings on this matter.*”

## Discussion

This paper draws on and utilises qualitative comments provided by bereaved relatives in order to shed an important light on patients’ and their relatives’ EoLC experiences in the acute hospital. The findings of this study report on five interconnected themes which impact on EoLC experiences from the perspective of bereaved relatives. Two themes relate to how care needs are met and how the hospital environment has a critical influence on EoLC experiences. The remaining three themes relate more to the interpersonal context including whether patients are treated with dignity and respect, the psychological, emotional and spiritual needs of patients and their family members and communication. The themes emerging from this study on EoLC are similar to those found in several other studies [13, 15, 16].

The Quality Standards for End-of-Life Care in Hospitals (3:67) state that there should be “*timely, clear and sensitive communication with each person, as appropriate, in respect of a diagnosis that s/he may be approaching or at end of life.*” To ensure this, there needs to be effective communication across and within the healthcare teams involved in the care of a person who is approaching end of life. Findings from this study, suggest the strong wish for clear, understandable, information and communication which is delivered in a timely and sensitive manner. Similar to other research [13, 14], the results of this study indicate that staff need to be more proactive in their communication to ensure clarity and more open discussions about prognosis and the possibility of dying so that wishes and preferences are met. Good communication centres on respecting patients’ dignity, autonomy and privacy whilst ensuring their wishes and needs are heard and understood. As reported by Mayland et al. [14] some relatives indicated that staff did not appear to have time to listen or to communicate with patients and their family members about their diagnosis or EoLC treatment plan and preferences. In the opinion of many relatives, communication practices in the acute hospital setting needed to be improved [13–15] so that healthcare professionals are skilled in respectful, timely and sensitive communication in all aspects of EoLC.

The fast pace of the acute hospital setting has been reported as being a major barrier to patient care at end of life [42–47]. Previous research has highlighted staff shortages and their perceived impact on meeting care needs at end of life [1, 15] as well as the need for more psychological and pastoral care and the opportunity to talk about death [15]. While relatives in our study also suggested that the quality of care they received was negatively impacted by perceived staff shortages, the majority were keen to stress the high skill level and dedication of the staff they came into contact with.

Psychosocial dimensions of EoLC coexist alongside symptom and pain management and are implicitly recognised by patients and their family members [48]. Findings in our study suggest that patients psychological, emotional and spiritual care needs were not always fully considered and responded to appropriately or in a timely manner. Provision of emotional and spiritual support to dying patients and their relatives are integral to good EoLC, with research suggesting that it can help moderate physical symptoms [24]. It is critical, therefore that hospitals review how they can better enhance the provision of emotional and spiritual support for patients and families. Research suggests that patients often feel that they are treated as a ‘number’ rather than as a patient [15]. Most relatives in this study reported being treated with dignity, respect and kindness, reflecting this desire for personalised attention which indicate that a personalised, compassionate approach to care at end of life had a lasting impact on relatives’ perception of care. It would appear that the significance of the emotional experience of EoLC provision cannot be underestimated [48].

Closely related and intertwined with the theme of dignity and respect was the impact of the hospital environment which has been repeatedly highlighted by patients and their families [16]. Patients often die in multi-occupancy rooms and noise levels in busy acute hospital wards negatively impacts on patient’s experiences at end of life [49]. While there is some debate around the use of single rooms for very ill older people at risk of falls when staff ratios are not adequate, the importance of patient preferences for care has been highlighted [16, 49]. Several studies have also noted the importance of single occupancy bed rooms for the prevention and spread of healthcare acquired infections [50, 51]. The importance of privacy at end of life has been reported in several studies [3, 16, 22, 49, 52]. Slatyer et al. [3:2170] in their study on the impact of a dedicated patient/family room for EoLC in an acute hospital setting found that caring for patients in this space enabled “more effective emotional support” and relieved stress and anxiety. Brereton et al. [49] reported privacy and proximity to loved ones as important factors at end of life. In our study, privacy was considered a key factor in influencing a patient’s end of life experience and the provision of care in a single occupancy bed room was a key factor for the majority of bereaved relatives. Access to family rooms with adequate facilities was also found to impact positively on that experience. Other studies have identified access to facilities for refreshments to be helpful [52].

In contrast to Harrop et al.’s [53] study, bereaved relatives in this study did not express feelings of guilt or trauma relating to difficult decisions which had to be made or the impact of the death experience suggesting that relatives in our study had a more positive EoLC experience. Of concern however, is that without access to a single room, patients and their family members reported being unable to spend private, uninterrupted, treasured time together. Furthermore, some relatives commented on the lack of information and support they received from staff regarding unrestricted visiting times and a lack of advice as to how they should communicate with the patient about the fact they were dying. Findings suggest that bereaved relatives may have unmet information needs which can contribute to feelings of ‘helplessness’ [15].

Research has found that relatives receive a variety of post-bereavement support depending on the location of death (at home, hospice, hospital) [15]. Studies have highlighted the positive impact of post-death supports on the grieving process for family members [15, 16, 53]. Holtslander et al. [54] reported that bereaved caregivers would like a continuity of support from the healthcare staff who had cared for their relative prior to their death and drew attention to the importance of this relationship. Relatives in our research frequently commented on the close relationships built between staff, patient and family and the therapeutic benefit of those relationships for them. The emotional labour, where staff actively manage relationships with patients and families is informally recognised as a key skill which shapes and influences a patient’s end-of-life journey [48]. Healthcare staff have also identified the need for a bereavement follow up service which would act to both deal with bereaved relatives need for counselling and information as well as elicit their views about the care they received [52]. Hospitals therefore, need to ensure standardised post-bereavement supports. The importance of preparing patients and families for death and providing contact and support post-bereavement cannot be over stated [16]. Bereaved relatives in our study highlighted the importance of receiving practical information about what happens following the death of a family member in hospital, including information on bereavement support.

Concern has been expressed about surveying this population, however other studies have noted the benefits [55, 56]. Bereaved relatives in our study shared detailed moving insights into their experiences of EoLC. The high response rate and considerable volume of qualitative data supports the premise and methodology of surveying bereaved relatives as part of a quality improvement process in acute hospitals. Germain et al. [56:482] reported that bereaved relatives benefited from participating in their research, they were given the opportunity to tell their ‘story’. While the sample size was small, nonetheless, the experience was described as ‘therapeutic’, highlighting the value of involving bereaved relatives in research on EoLC. Relatives in our study expressed similar views, stating the experience as being supportive and offering them an opportunity to express their views about care at end of life and others used it as an opportunity to express their gratitude to the hospital and staff.

Several studies have reported findings from open-ended questions that were part of a larger survey on EoLC [13, 15, 57]. Seeking the views of bereaved relatives in a sensitive, structured and safe way, should be considered by all acute hospitals, to ascertain the quality of care at end of life and to support the development of quality EoLC in the acute hospital setting.

While the bereaved relatives in this study reported many positive elements about the EoLC they received in the two hospitals they also identified improvements that could be made to enhance care for future patients and their families. Listening to the ‘voices’ of bereaved relatives is critical to quality improvement in EoLC.

### Strengths, limitations and implications for research

The main strength of these findings is the high response rate resulting in an unanticipated volume of qualitative ‘free’ text, proving rich data on experiences of EoLC in the two hospitals. The use of open ended, free text questions facilitated the expression of subjective experiences and provided rich, powerful insights which could not have been captured through using closed questions alone. In addition, this is the largest survey of bereaved relatives conducted in two adult Irish acute hospitals. The study has several limitations including the use of proxy in representing the patient view. While the reliability of proxy reporting has been questioned by some [58–60], others express confidence about its reasonable validity and correspondence with patient’s views [13, 61, 62]. Findings are representative of those who responded and may not be representative of those relatives who chose not to take part in the study. Whilst the themes mirror findings from international research, they may not be generalised to represent the care provided in other acute hospital settings nationally or internationally.

## Conclusion and implication for research

Acute hospitals are fast paced environments, predominantly focussed on diagnosis, treatment and cure of serious illness and the care and management of chronic illness. However, the care of those with a life-limiting illness is also an important role as is the care of those people who may die in this setting. Given this, acute hospitals need to ensure that patients and their relatives receive high quality care at end of life and have their individual care needs met. Seeking the views of bereaved relatives should be considered by all hospitals and healthcare settings to ascertain the quality of care at end of life. This study has contributed to our understanding and knowledge of what is important to people at end of life, what good EoLC looks like and where care can be improved from the perspective of bereaved relatives thus, informing policy and practice and enabling hospitals to direct and inform quality improvement.

## Abbreviations

EoLC: End-of-life Care
MDT: Multi-disciplinary Team
